# A proof for loop-law constraints in stoichiometric metabolic networks

**DOI:** 10.1186/1752-0509-6-140

**Published:** 2012-11-12

**Authors:** Elad Noor, Nathan E Lewis, Ron Milo

**Affiliations:** 1 Department of Plant Sciences, 234 Herzl st., Weizmann Institute of Science, Rehovot 76100, Israel; 2 Department of Bioengineering, 9500 Gilman Drive, University of California San Diego, La Jolla, CA 92093-0412, USA; 3 Wyss Institute for Biologically Inspired Engineering and Department of Genetics, 77 Avenue Louis Pasteur, Harvard Medical School, Boston, MA 02115, USA

**Keywords:** Thermodynamics, Gordan’s theorem, Metabolism

## Abstract

**Background:**

Constraint-based modeling is increasingly employed for metabolic network analysis. Its underlying assumption is that natural metabolic phenotypes can be predicted by adding physicochemical constraints to remove unrealistic metabolic flux solutions. The loopless-COBRA approach provides an additional constraint that eliminates thermodynamically infeasible internal cycles (or loops) from the space of solutions. This allows the prediction of flux solutions that are more consistent with experimental data. However, it is not clear if this approach over-constrains the models by removing non-loop solutions as well.

**Results:**

Here we apply Gordan’s theorem from linear algebra to prove for the first time that the constraints added in loopless-COBRA do not over-constrain the problem beyond the elimination of the loops themselves.

**Conclusions:**

The loopless-COBRA constraints can be reliably applied. Furthermore, this proof may be adapted to evaluate the theoretical soundness for other methods in constraint-based modeling.

## Background

**Figure 1:**
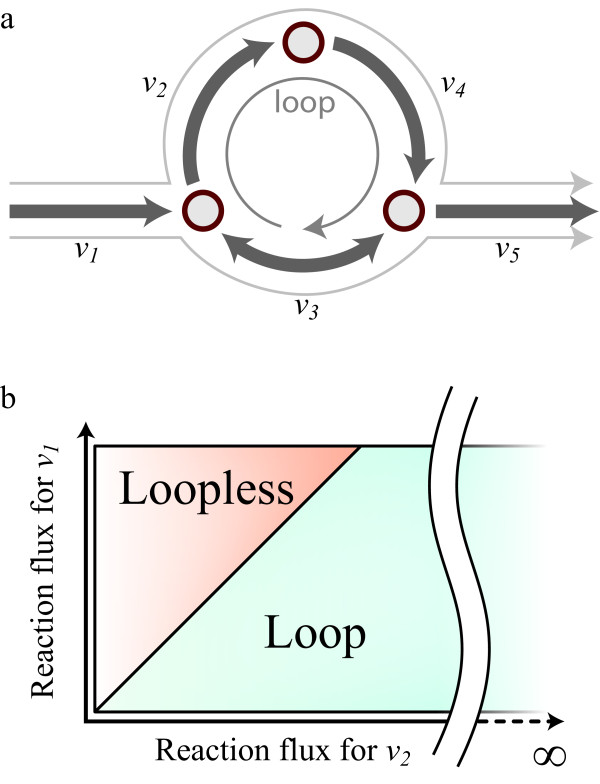
**Loop law constraints on metabolic networks.(a)** Metabolic network reconstructions frequently have sets of reactions that cycle all metabolites internally. The fluxes of these reactions are therefore unconstrained. **(b)** Metabolic network solutions are found within a convex space, which is enclosed by known constraints on metabolite inputs, outputs, and known fluxes. Loops result in unconstrained dimensions in the solution space (blue). By implementing loopless-COBRA constraints, all loop-containing solutions are removed, leaving only solutions that do not contain loops (orange).

Constraint-based modeling has become a successful framework for the analysis of large and complex stoichiometric biochemical networks [1]. The underlying concept of this framework is that one can use the stoichiometry of each reaction in a reconstructed metabolic network [2,3] and known bounds on reaction fluxes to compute metabolic flux for each reaction. These predictions represent allowable steady-state metabolic flux distributions in a cell under a given growth condition. Some example constraints include mass balance and metabolite uptake rates. One set of constraints that has been more challenging to implement are those associated with thermodynamic limitations. Without thermodynamic constraints, non-physical fluxes can be computed for some metabolic reactions if they produce an internal cycle (Figure 1a). Such cycles of reactions violate a “loop law” that is analogous to Kirchhoff’s second law for electrical circuits, as discussed previously by Beard et al. [4]. Many approaches have successfully constrained these loops using known flux directionality [5], energy-balance equations [4], and known [6-11] or predicted [12] thermodynamic parameters. Loops have also been indirectly removed by minimizing network flux [6,13-15], or by coupling flux to enzyme synthesis costs [16]. 

A new approach, called loopless-COBRA, was recently presented [17]. Unlike previous loop-removal algorithms, this method does not necessitate extra inputs or data, such as metabolite concentrations or thermodynamic parameters. Basically, this method imposes the second law of thermodynamics by using a mixed-integer linear programming (MILP) approach to constrain flux solutions so that they obey the loop law. Thus, flux solutions from this method are all within the portion of the flux space that is devoid of loops (Figure 1b). Excitingly, the loop removal improved the consistency of the simulation results [17] with experimental data [18]. Specifically, it provides more realistic flux values for reactions that normally contribute to loops in a model. Otherwise, flux predictions for such reactions would usually have to be ignored in any subsequent analysis.

The loopless-COBRA method was shown to work in various scenarios, and the paper that presented the approach provides an explanation for why this method works. However, there is no mathematical proof for its formulation as an optimization problem. Specifically, it does not demonstrate that the additional MILP constraints do not over-constrain the problem and eliminate some non-loop containing solutions. Since constraint-based methods attempt to only eliminate impossible *in silico* phenotypes (i.e., steady-state flux distributions that the cell cannot maintain), it is important to verify that solutions representing real phenotypes are not removed by accidentally over-constraining the problem.

Here, we address this issue by presenting a mathematical proof for the completeness and soundness of the loopless-COBRA method, thereby adding fundamental support and rigorous proof for the constraints presented by Schellenberger et al. [17].

## Results and discussion

### Formal definition of loop-law constraints

In loopless-COBRA, the constraints added to the linear problem are:

$$\begin{array}{lll} & \;\;\;G_{i}<0\;\text{for all}\;v_{i}>0\; & \;\\ & \;\;\;G_{i}>0\;\text{for all}\;v_{i}<0\; & \;\\ & \;\;\;G_{i}\in R\;\text{for all}\;v_{i}=0\; & \;\\ & N_{\mathrm{int}}G=0\; & \; \end{array}$$

where *v*_*i*_ are the flux variables and *N*_*int*_ is the null-space matrix of *S*_*int*_(the stoichiometric matrix of internal reactions). The third constraint ( $G_{i}\in R$) is not actually a constraint, but a way to say that *G*_*i*_ can have any value if *v*_*i*_ = 0. We can rewrite all these constraints succinctly as:

(1) $$\exists G\in R^{n}\;\text{s.t.}\;\text{null}(S_{\mathrm{int}})\cdot G\;=\;0\wedge \left(\forall i\;\text{sign}(G_{i})\;=\;-\text{sign}(v_{i})\vee v_{i}\;=\;0\right)$$

### Formal definition of loops

In order to prove that this constraint eliminates loops (and only loops), we must first find a mathematical formulation for a loop, using the same notation as above. We thus define a loop as a nonzero vector $x\in R^{n}$ which satisfies the mass-balance equation for the internal reactions, i.e. *S*_*int*_ · *x* = 0. This means that although there is a nonzero net flux in some of the reactions, overall, the internal network is at steady-state (an obvious violation of the second law of thermodynamics). It is important to point out, that this equation for defining loops must not be confused with the steady-state assumption commonly used in flux balance analysis models, namely *S*·*v* = 0, where the *full* stoichiometric matrix (*S*) is used.

According to this definition, a flux distribution ( $v\in R^{n}$) will contain a loop if and only if there exists a vector $x\in R^{n}\setminus \{0\}$ which is consistent with the flux directions in *v* (i.e. *x*_*i*_ is either zero or has the same sign as *v*_*i*_) and is itself a loop (i.e. *S*_*int*_ · *x* = 0). Formally, *v* has a loop if and only if:

(2) $$\exists x\in R^{n}\setminus \{0\}\;\text{s.t.}\left(\forall i\;\text{sign}(x_{i})\in \{\text{sign}(v_{i}),0\}\right)\wedge S_{\mathrm{int}}\cdot x=0$$

We have now finished laying the groundwork for our mathematical proof that loopless-COBRA is sound and complete. In order to do that, we are left only to show that Equation 1 is satisfiable if and only if Equation 2 is unsatisfiable (in other words, there are no loops).

### Gordan’s theorem

We start our proof by quoting Gordan’s theorem: For all $A\in R^{m\times n}$ exactly one of the following two statements is true:

$$\begin{array}{lll} & (a)\;\exists x\in R_{+}^{n}\setminus \{0\}\;\;\text{s.t.}\;\;\text{Ax}=0\; & \;\\ & (b)\;\exists y\in R^{m}\;\;\text{s.t.}\;\;A^{⊤}y>0\; & \; \end{array}$$

We will show that statement (*a*) in Gordan’s theorem is equivalent to having a loop (Equation 2) and statement (*b*) is equivalent to the MILP constraints used by loopless-COBRA (Equation 1). After doing so, we would easily reach the conclusion of the proof.

As a guidance for the following sections, one can see that statement (*a*) already resembles Equation 2 if we define *A* = *S*_*int*_. The only difference is that *x* is constrained to have only non-negative values (note the ‘+’ in $R_{+}^{n}$), instead of being consistent with the sign of *v*. Corollaries 1 and 2 will show how we can overcome this discrepancy by defining *A* in a slightly different way.

**Figure 2:**
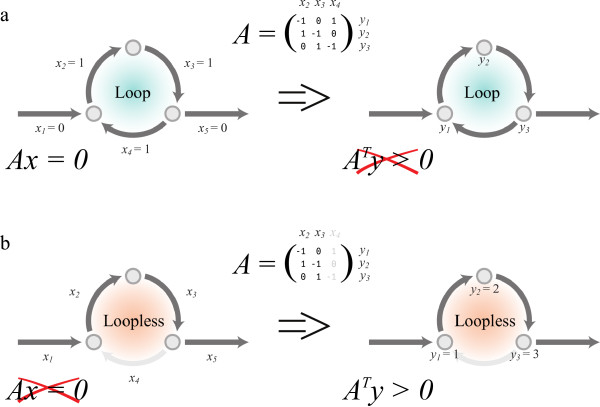
**Illustrative example for Corollary 2.** This example shows a small network with 3 internal reactions (*x*_2−4_). The flux directions were chosen according to the direction of the arrows. The matrix *A* is the internal stoichiometric matrix. **(a)** A flux distribution is shown where all 3 internal reactions are active and form a loop. Therefore there is a solution (*x*_2_ = *x*_3_ = *x*_4_ = 1) for the mass balance equation *Ax* = 0. In this case, no solution exists for *A*^⊤^*y* > 0. Therefore this flux distribution will be eliminated by loopless-COBRA. **(b)** A loopless flux distribution, in this case *x*_4_is not active. There is no solution for *Ax* = 0(except for the trivial solution *x* = 0). Gordan’s theorem claims that there must be a solution for *A*^⊤^*y* > 0, e.g. the one shown in the figure. Thus, loopless-COBRA will not eliminate any such flux distributions.

At first glance, statement (*b*) might look unrelated to Equation 1. However, in the last part of our proof, we show that choosing $G\;\in \;R^{n}$, which satisfies null(*S*_*int*_)*G* = 0, is the same as choosing $y\;\in \;R^{m}$ and then taking $G=S_{\mathrm{int}}^{⊤}\cdot y$. Only for sake of understanding the algebra, one can think of *y* as the vector of formation Gibbs energies, and of *G* as the vector of reaction Gibbs energies. The rest of the proof, like for statement (*a*), deals with adjusting *A* to fit with the non-positive values in *v*. The toy example in Figure 2 shows how Gordan’s theorem corresponds to having or not having a loop, for a network with 3 compounds and 3 internal reactions.

### Corollary 1

For all $A\in R^{m\times n}$ and *d* ∈ {−1,0,1}^*n*^ exactly one of the following two statements is true:

$$\begin{array}{c} (1a)\;\exists x\in R^{n}\setminus \{0\}\;\text{s.t.}\;\left(\forall i\;\text{sign}(x_{i})\in \{d_{i},0\}\right)\wedge \text{Ax}=0\\ (1b)\;\exists y\in R^{m}\;\;\text{s.t.}\;\left(\forall i\;\text{sign}{\left(A^{⊤}y\right)}_{i}=d_{i}\vee d_{i}=0\right) \end{array}$$

#### Proof

First, define a new matrix Â that is the same as *A*, without the columns corresponding to *d*_*i*_ = 0 and where columns corresponding to *d*_*i*_ = −1 are multiplied by −1. Statement (1*a*) is true for *A* if and only if (*a*) is true for Â. The forward direction is easily shown by removing the zeros from *x*, where *d*_*i*_ = 0, and negating values corresponding to *d*_*i*_ = −1 (as previously done for *A*). Reversing this process (i.e. taking a positive solution for $Â\hat{x}=0$, adding back the zeros and negating the same values) shows the other direction is true as well.

Likewise, statement (1*b*) for *A* is true if and only if (*b*) is true for Â, since columns with *d*_*i*_ = −1 are negated in Â and thus sign ${\left({Â}^{⊤}y\right)}_{i}=1$. Columns with *d*_*i*_ = 0 have no other constraints in (1*b*) and the same goes for (*b*) since they are removed from Â.

Therefore, Corollary 1 is directly derived from Gordan’s theorem. □

Since constraint-based models usually use a vector of real values ( $v\in R^{n}$) to represent the flux distribution, we subsequently change the formulation of the Corollary slightly to match.

### Corollary 2

For all $A\in R^{m\times n}$ and $v\in R^{n}$, exactly one of the following two statements is true:

$$\begin{array}{c} (2a)\;\;\;\;\exists x\in R^{n}\setminus \{0\}\;\;\text{s.t.}\;\left(\forall i\;\text{sign}(x_{i})\in \{\text{sign}(v_{i}),0\}\right)\wedge \mathrm{Ax}=0\\ (2b)\;\;\;\;\exists y\in R^{m}\;\;\;\text{s.t.}\;\left(\forall i\;\text{sign}{\left(A^{⊤}y\right)}_{i}=-\text{sign}(v_{i})\vee v_{i}=0\right) \end{array}$$

#### Proof

Defining *d*_*i*_ ≡ sign(*v*_*i*_), we get this directly from Corollary 1. Note that −sign(*v*_*i*_) can be used in (2b), since the existence of *y* is equivalent to the existence of −*y*. □

This adjustment now allows us to apply Corollary 2 to constraint-based problems and show that it eliminates loops (see example in Figure 2). In order to avoid any confusion, we point out that a solution for *Ax* = 0 is considered a loop only if *A* is the stoichiometric matrix of *internal* reactions. This should not be mistaken as the steady-state mass-balance equation which looks exactly the same, except that *A* contains both internal and external reactions.

### Corollary 3

Adding the following constraint:

$$\exists y\in R^{m}\;\text{s.t.}\;\left(\forall i\;\text{sign}{\left(A^{⊤}y\right)}_{i}=-\text{sign}(v_{i})\;\vee \;v_{i}=0\right)$$

is equivalent to eliminating all loops in a flux distribution *v*.

#### Proof

Using Corollary 2, all that must be shown is that statement (2a) is equivalent to having a loop. This is apparent since *x* is a vector in the null-space of *A* (i.e., a loop) and is consistent with the flux direction of *v* in each of its nonzero reactions. □

Note that the trivial case *v* = 0 can still be a solution and it should be explicitly avoided if necessary.

### Applying Corollary 3 in loopless-COBRA

The added constraints in loopless-COBRA [17] are slightly different than in Corollary 3, namely:

$$\begin{array}{ll} \exists G\;\in \;R^{n}\;\;\text{s.t.}\;\;\text{null}(A)\;\cdot \;G\;=\;0\;\wedge \;\left(\forall i\;\text{sign}(G_{i})\;=\;-\text{sign}(v_{i})\;\vee \;v_{i}\;=\;0\right) & \; \end{array}$$

We claim here that both formulations are equivalent. The fundamental theorem of linear algebra states that the nullspace, null(*A*), is the orthogonal complement of the row space, image(*A*^⊤^). Therefore, we can say that null(*A*)·*G* = 0 if and only if *G* ∈ image(*A*^⊤^), so we can rewrite the constraint above as:

$$\exists G\in \text{image}(A^{⊤})\;\text{s.t.}\;\left(\forall i\;\text{sign}(G_{i})=-\text{sign}(v_{i})\;\vee \;v_{i}=0\right)$$

which is obviously equivalent to the constraint in Corollary 3.

## Conclusions

Our results prove that the constraints proposed by Schellenberger, et al. [17] eliminate all flux solutions with loops and nothing more. This alleviates the concern as to if the loopless-COBRA constraints might eliminate true flux states. Furthermore, values in *G* are analogous to the change in Gibbs energy (*Δ*_*r*_*G*) of the reactions [17], and *y* values are analogous to the chemical potentials (or formation energies, *Δ*_*f*_*G*) of the compounds themselves. Since in most cases, there are fewer compounds than reactions (*m* < *n*), we believe that it is convenient and intuitive to use the new formulation.

In conclusion, this proof provides theoretical credibility for the loopless-COBRA constraint. However, as with any algorithmic MILP implementation, care must still be taken with respect to numerical limitations and the convergence of the optimization algorithm.

Lastly, we believe this proof may be extended to similar methods addressing loop elimination. We also hope that similar proofs will appear for other methods, since more rigorous mathematical treatments are needed in many published algorithms in computational biology to prove or disprove their correctness.

## Competing interests

The authors declare that they have no competing interests.

## Authors’ contributions

EN conceived of the study, participated in its design and coordination, and helped to draft the manuscript. NL participated in the design of the study and drafted the manuscript. RM participated in its design, coordination, and drafting the manuscript. All authors read and approved the final manuscript.
